# Phloroglucinol as a Potential Candidate against *Trypanosoma congolense* Infection: Insights from In Vivo, In Vitro, Molecular Docking and Molecular Dynamic Simulation Analyses

**DOI:** 10.3390/molecules27020469

**Published:** 2022-01-12

**Authors:** Nasirudeen Idowu Abdulrashid, Suleiman Aminu, Rahma Muhammad Adamu, Nasir Tajuddeen, Murtala Bindawa Isah, Isa Danladi Jatau, Abubakar Babando Aliyu, Mthokozisi Blessing Cedric Simelane, Elewechi Onyike, Mohammed Auwal Ibrahim

**Affiliations:** 1 Department of Biochemistry, Ahmadu Bello University, Zaria 810241, Nigeria; abdulrashidmuhammadnasir@gmail.com (N.I.A.); suleimanaminu@abu.edu.ng (S.A.); elewechionyike@gmail.com (E.O.); 2 Department of Biotechnology, School of Engineering and Technology, Sharda University, Greater Noida 201310, India; turanjoji84@gmail.com; 3 Department of Chemistry, Ahmadu Bello University, Zaria 810241, Nigeria; ntajuddeen@yahoo.com (N.T.); aliyubabando@gmail.com (A.B.A.); 4 Department of Biochemistry, Umaru Musa Yar’adua University, Katsina 820241, Nigeria; isah.murtala@umyu.edu.ng; 5 Department of Veterinary Parasitology and Entomology, Ahmadu Bello University, Zaria 810241, Nigeria; mail4idjatau@gmail.com; 6 Department of Biochemistry, University of Johannesburg, Johannesburg 2001, South Africa; msimelane@uj.ac.za

**Keywords:** anemia, phloroglucinol, phospholipase A_2_, sialidase, *Trypanasoma congolense*, molecular dynamics simulation, molecular docking

## Abstract

Sub-Saharan Africa is profoundly challenged with African Animal Trypanosomiasis and the available trypanocides are faced with drawbacks, necessitating the search for novel agents. Herein, the chemotherapeutic potential of phloroglucinol on *T. congolense* infection and its inhibitory effects on the partially purified *T. congolense* sialidase and phospholipase A_2_ (PLA_2_) were investigated. Treatment with phloroglucinol for 14 days significantly (*p* < 0.05) suppressed *T. congolense* proliferation, increased animal survival and ameliorated anemia induced by the parasite. Using biochemical and histopathological analyses, phloroglucinol was found to prevent renal damages and splenomegaly, besides its protection against *T. congolense*-associated increase in free serum sialic acids in infected animals. Moreover, the compound inhibited bloodstream *T. congolense* sialidase via mixed inhibition pattern with inhibition binding constant (Ki) of 0.181 µM, but a very low uncompetitive inhibitory effects against PLA_2_ (Ki > 9000 µM) was recorded. Molecular docking studies revealed binding energies of −4.9 and −5.3 kcal/mol between phloroglucinol with modeled sialidase and PLA_2_ respectively, while a 50 ns molecular dynamics simulation using GROMACS revealed the sialidase-phloroglucinol complex to be more compact and stable with higher free binding energy (−67.84 ± 0.50 kJ/mol) than PLA_2_-phloroglucinol complex (−77.17 ± 0.52 kJ/mol), based on MM-PBSA analysis. The sialidase-phloroglucinol complex had a single hydrogen bond interaction with Ser453 while none was observed for the PLA_2_-phloroglucinol complex. In conclusion, phloroglucinol showed moderate trypanostatic activity with great potential in ameliorating some of the parasite-induced pathologies and its anti-anemic effects might be linked to inhibition of sialidase rather than PLA_2_.

## 1. Introduction

African animal trypanosomiasis (AAT) is a parasitic disease caused by a wide range of *Trypanosoma* species in sub-Saharan African countries that largely represses economic development in the region by affecting livestock industries [1]. Unfortunately, drug discovery research for AAT is limited compared with the human form of the disease [2]. Among the species causing AAT, *Trypanosoma congolense* is the most pathogenic leading to debilitating disease in cattle and other domestic animals [3]. As an extracellular parasite, *T. congolense* remains confined to the host vascular system during the course of infection and subsequently attaches itself to the host’s erythrocyte membrane, resulting in damage at the adhesion sites [4]. The activities of the parasite within the host ultimately lead to pathologies such as organ degeneration, fever, loss of weight and severe anemia which could ultimately lead to the death of the infected animals [5,6]. Considering the negative effects of the parasite and the numerous drawbacks of the available antitrypanosomal drugs [7], urgent attention is needed by the scientific communities to discover new drug candidates.

In recent years, scientific attention has keenly focused on the trypanosome induced-anemia because it is considered as the most prominent pathological feature and symptom of the infection [8]. The *T. congolense*-induced anemia is mainly mediated by sialidase (EC 3.2.1.18) and phospholipase A_2_ (PLA_2_) (EC 3.1.1.4) released by the parasite during infection [9,10,11]. As an important molecule, sialic acid cannot be synthesized by the parasite de novo and therefore the parasite obtains it from the surface of the host’s erythrocyte through the action of sialidase. The removal of sialic acid exposes the *β*-galactosyl residue on the host’s erythrocyte, leading to the activation of macrophages, erythrophagocytosis and anemia [12]. Similarly, African trypanosomes do not have the capacity for de novo fatty acid synthesis but again, scavenge it from the surface of the host’s erythrocytes using PLA_2_ [13]. The action of the trypanosomal PLA_2_ leads to anemia by destructing the host erythrocyte membrane thereby releasing high amount of free fatty acids that exceed the binding capacity of albumin. The released fatty acids are used by the parasite to meet its lipid requirements [14]. Compounds targeting sialidase and PLA_2_ have shown promising activities against the parasite and other diseases [15,16,17,18]. Indeed, we have also shown that some compounds have trypanostatic activity and prevented the release of free serum sialic acid from the host’s erythrocyte membrane through the inhibition of sialidase [6,19,20,21].

**Figure 1:**
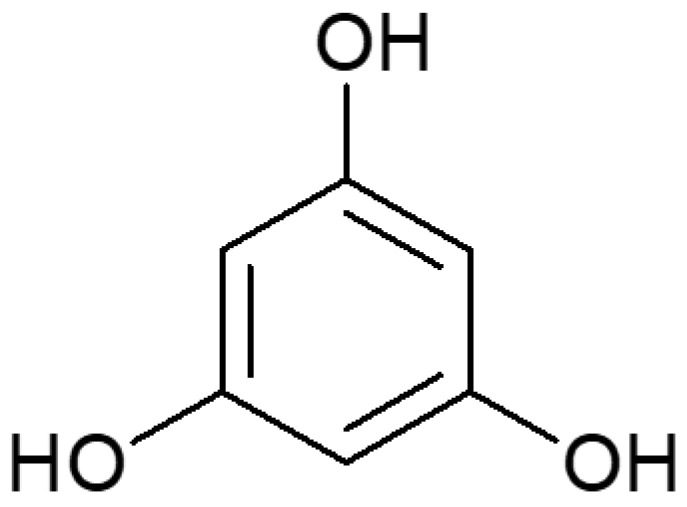
Structure of phloroglucinol.

African medicinal plants are well reputed to contain a repertoire of compounds with great potentials to serve as antitrypanosomal candidates [22]. Among the African antitrypanosomal plants, *Khaya senegalensis* is one of the most promising and highly investigated plant [10,22,23,24]. Previously, we reported the antitrypanosomal activity of a phloroglucinol-rich fraction of an extract from the stem bark of the plant where it eliminated *T. brucei brucei* from the bloodstream of infected animals at 200 mg/kg BW [10]. Unfortunately, the fraction was highly toxic to uninfected (normal) animals which warranted further combination therapy with vitamins C and E aimed at mitigating the toxicity of the fraction whilst maintaining the potent antitrypanosomal activity [25,26]. Such approach did not yield a positive outcome. Therefore, in our quest to discover novel and therapeutically viable trypanocide from the fraction, we investigated the in vivo chemotherapeutic effect of pure phloroglucinol (Figure 1) against *T. congolense* infection and its effect on anemia amelioration, as well as organ damage during the disease. Furthermore, in order to gain mechanistic insights into anemia amelioration, we investigated the in vitro inhibitory effects of the compound on bloodstream *T. congolense* sialidase and PLA_2_ in addition to molecular dynamics simulation of the compound in complex with the two enzymes.

## 2. Results

**Figure 2:**
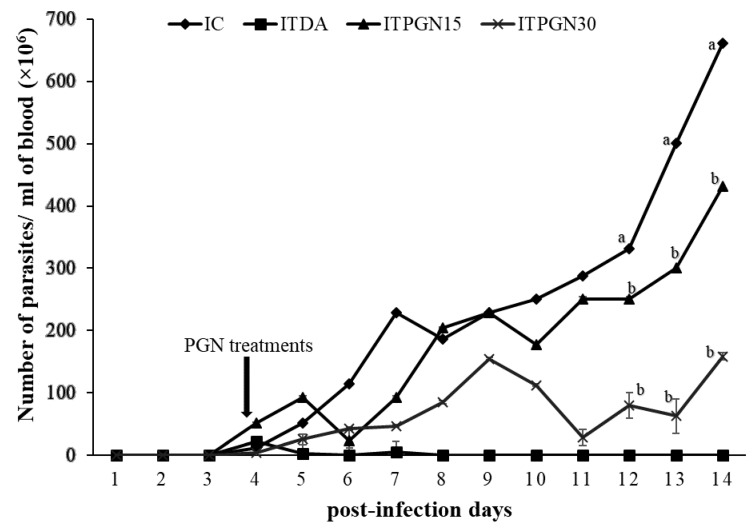
Evaluation of parasitemia in *T. congolense*-infected rats treated with different doses of phloroglucinol. All data are presented as mean ± standard deviation of seven rats. Dunnett posthoc test was used to analyze the data following ONE-WAY ANOVA. IC was used as control. Values with different subscripts are considered statistically significant at *p* < 0.05. IC = infected control, ITPGN15 = Infected + 15 mg/kg BW PGN, ITPGN30 = Infected + 30 mg/kg PGN, ITDA = Infected + 3.5 mg/kg BW DA.

**Figure 3:**
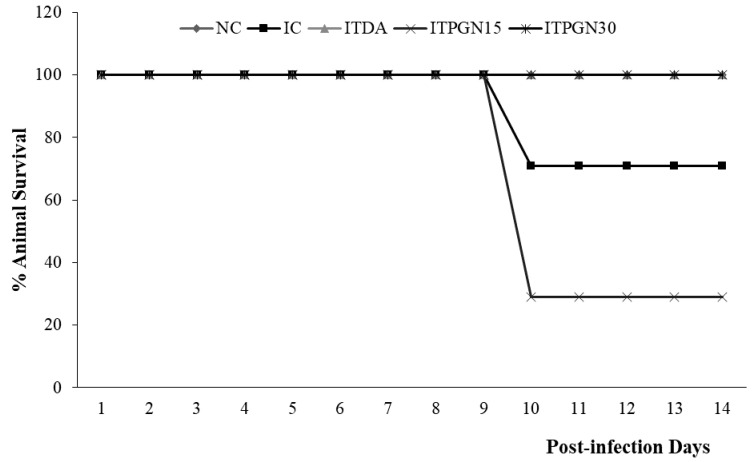
Cessation of mortality in *T. congolense*-infected rats treated with different doses of phloroglucinol. NC = Normal control, IC = Infected control, ITPGN15 = Infected + 15 mg/kg BW PGN, ITPGN30 = Infected + 30 mg/kg BW PGN, ITDA = Infected + 3.5 mg/kg BW DA.

Successful inoculation of *T. congolense* in experimental animals was ascertained by the appearance of parasite on day 4 post infection (pi). There was a rapid increase in parasite load observed in the infected animals which was sustained throughout the experimental period. It was also observed that between days 4 and 6 pi, treatment with phloroglucinol did not significantly (*p* > 0.05) alter the course of parasitemia as both IC, ITPGN15 and ITPGN30 showed similar parasitemia pattern (Figure 2). As treatment progressed, treatment with phloroglucinol significantly (*p* < 0.05) retarded the proliferation of the *T. congolense* parasites in the infected animals and the effect was more pronounced from days 12 to 14 pi. However, phloroglucinol treatment at all doses did not eliminate the parasites throughout the period of experiment, whereas parasite was not detected in the ITDA group following treatment (Figure 2). Increase in survival of the *T. congolense*-infected animals was also monitored following treatment with phloroglucinol. Mortality in the IC and ITPGN15 groups began on the 10th day pi, reducing the % survival of the animals to 60% and 25% respectively, which was maintained for the remaining 4 days of the experiment (Figure 3). An interesting finding was the ability of 30 mg/kg BW phloroglucinol to maintain 100% survival of the infected animals (Figure 3) and similar trend was observed in ITDA group (Figure 3).

**Figure 4:**
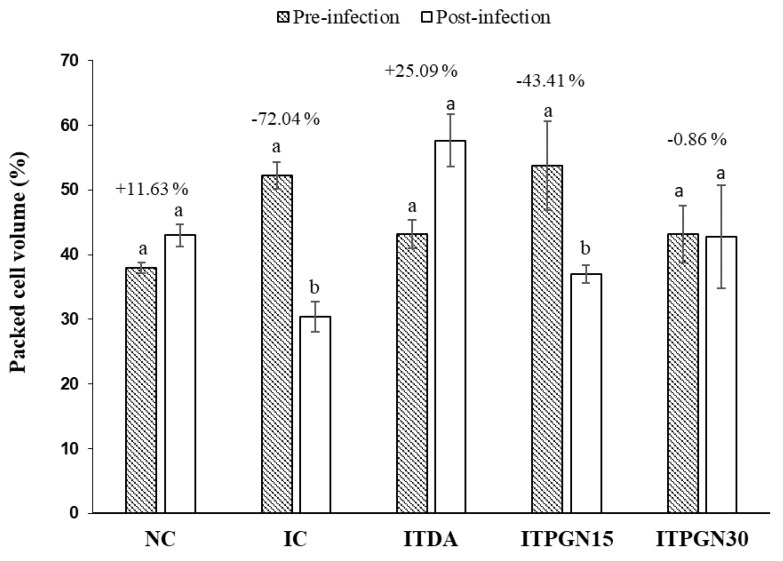
Assessment of anemia progression in *T. congolense*-infected rats treated with different doses of phloroglucinol. All data are presented as mean ± standard deviation of seven rats. Data was analyzed using paired sample t. test within group. Bars with different alphabets are considered statistically significant at *p* < 0.05. NC = Normal control, IC = Infected control, ITPGN15 = Infected + 15 mg/kg BW PGN, ITPGN30 = Infected + 30 mg/kg BW PGN, ITDA = Infected + 3.5 mg/kg BW DA.

Infection with *T. congolense* was accompanied with anemia, as evidently observed with drastic fall in final PCV of the infected untreated rats when compared with their baseline PCV value (Figure 4). There was a significant (*p* < 0.05) improvement in PCV value in the animals treated with 30 mg/kg BW phloroglucinol with % change in PCV of −0.86% showing anemia-amelioration potential compared to −72% in IC group (Figure 4). Moreover, the PCV of the animals in the NC and ITDA groups increased on the 14th day pi compared to day 0 (Figure 4).

**Figure 5:**
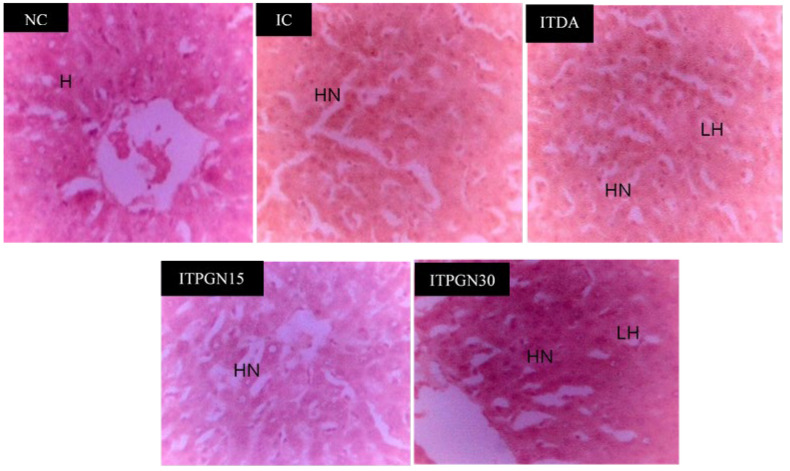
Liver histopathology of *T. congolense*-infected rats treated with different doses of phloroglucinol. H = Hepatocytes Normal, HN = Hepatocellular Necrosis, LH = Lymphocyte Hyperplasia. NC = Normal control, IC = Infected control, ITPGN15 = Infected + 15 mg/kg BW PGN, ITPGN30 = Infected + 30 mg/kg BW PGN, ITDA = Infected + 3.5 mg/kg BW DA.

**Figure 6:**
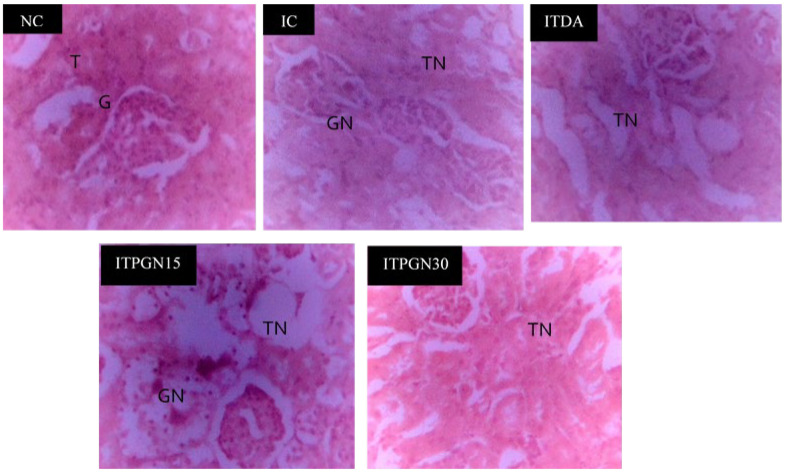
Kidney histopathology of *T. congolense*-infected rats treated with different doses of phloroglucinol. T = Normal Tubules, G = Normal Glomerulus, GN = Glomerular Necrosis, TN = Tubular Necrosis. NC = Normal control, IC = Infected control, ITPGN15 = Infected + 15 mg/kg BW PGN, ITPGN30 = Infected + 30 mg/kg BW PGN, ITDA = Infected + 3.5 mg/kg BW DA.

The potential role of phloroglucinol towards hepatic and renal damage in addition to organs enlargement was investigated by determining the levels of serum biomarkers as well as relative organs weight in the experimental animals (Table 1). Compared to the NC group, the IC group had a significant (*p* < 0.05) increase in serum levels of urea and creatinine with concomitant increase (*p* > 0.05) in serum activities of ALT and AST (Table 1). However, it was only the 30 mg/kg body weight (BW) phloroglucinol treatment that showed some positive tendencies toward ameliorating the trypanosome-induced perturbations to these biochemical indices, especially the renal parameters. Similarly, histopathology of the liver indicated that hepatic necrosis in the IC group was not reversed in the phloroglucinol-treated groups (Figure 5) but the trypanosome-induced glomerular necrosis was ameliorated by the 30 mg/kg BW of phloroglucinol (Figure 6), indicating positive ameliorative effects toward the kidney. Furthermore, renal hypertrophy, splenomegaly and brain enlargement observed in the IC group were ameliorated by phloroglucinol, especially at 30 mg/kg BW where the differences were significant (*p* < 0.05) (Table 1). Conversely, hepatomegaly was not significantly (*p* > 0.05) affected by the phloroglucinol at all dosages. On the other hand, the level of serum FSA was mildly elevated in IC group following infection with *T. congolense* but only 15 mg/kg BW phloroglucinol and 3.5 mg/kg BW diminazine aceturate significantly (*p* > 0.05) reduced the level of FSA (Table 1).

**Figure 7:**
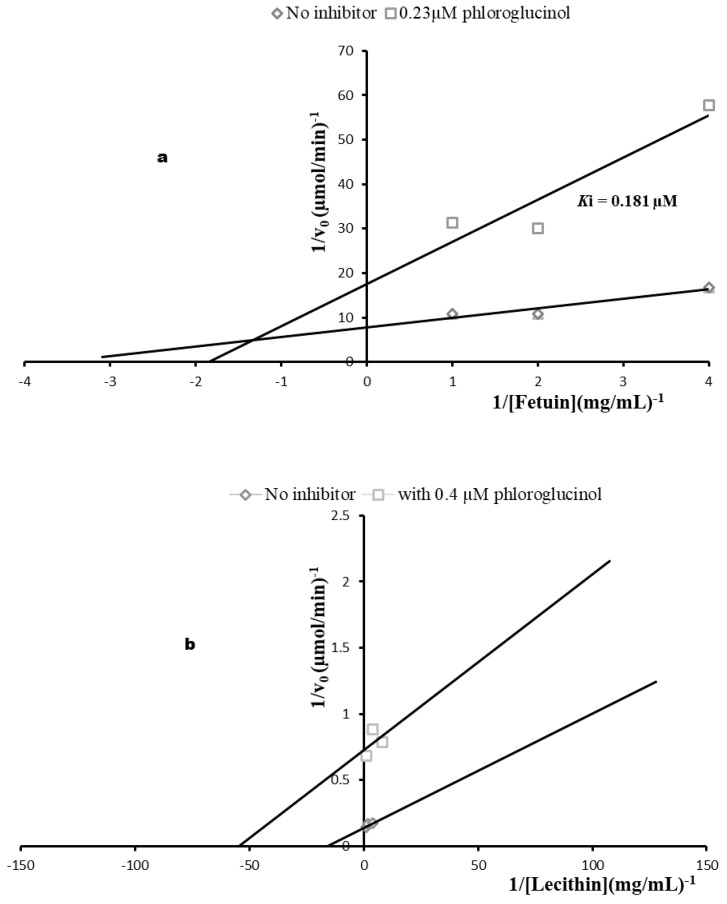
Double reciprocal plot of *T. congolense* sialidase (**a**) and PLA_2_ (**b**) inhibitions using phloroglucinol. All data are presented as mean ± standard deviation of three independent experiments. Ki = Inhibition constant.

The observation of the positive effect of phloroglucinol on *T. congolense* induced anemia prompted us to investigate its in vitro inhibitory effects on the parasite sialidase and PLA_2_. The compound inhibited sialidase with a Ki of 0.181 µM via a mixed inhibition pattern (Figure 7a) whereas Ki of 96,714.0 µM was computed against the *T. congolense* PLA_2_ and the mechanism of inhibition was uncompetitive (Figure 7b).

**Figure 8:**
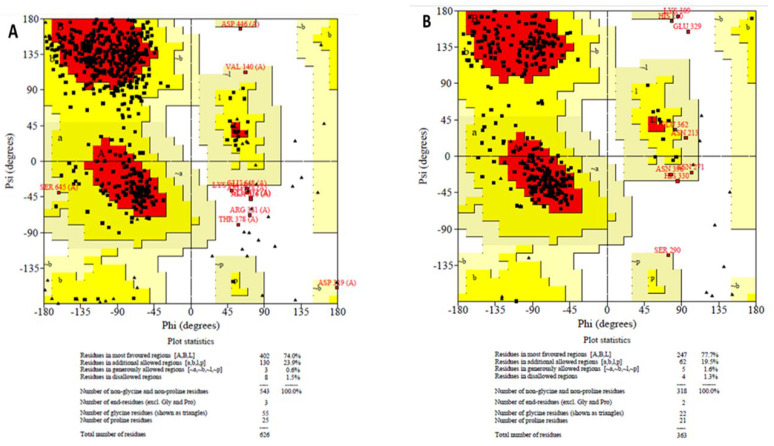
Ramachandran plot of modelled trypanosomal sialidase (**A**) and PLA_2_ (**B**) showing amino acid residue regions.

**Figure 9:**
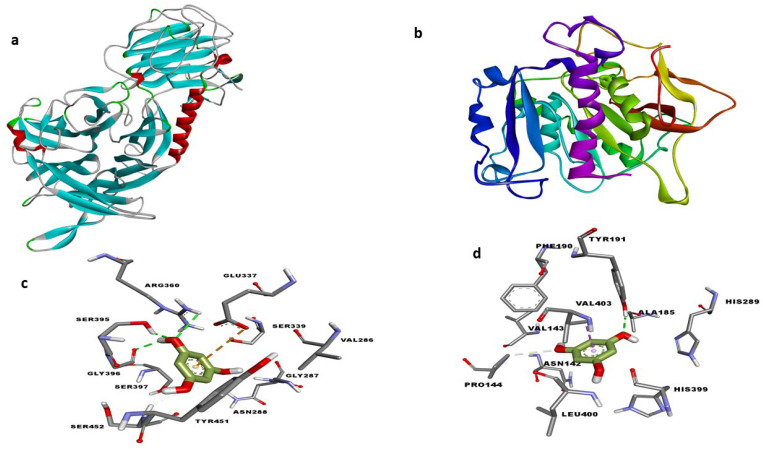
Homology models of trypanosomal sialidase (**a**) and PLA_2_ (**b**) after 50 ns molecular dynamics simulation and 3D molecular docking interactions of phloroglucinol with the trypanosomal sialidase (**c**) and PLA_2_ (**d**).

**Figure 10:**
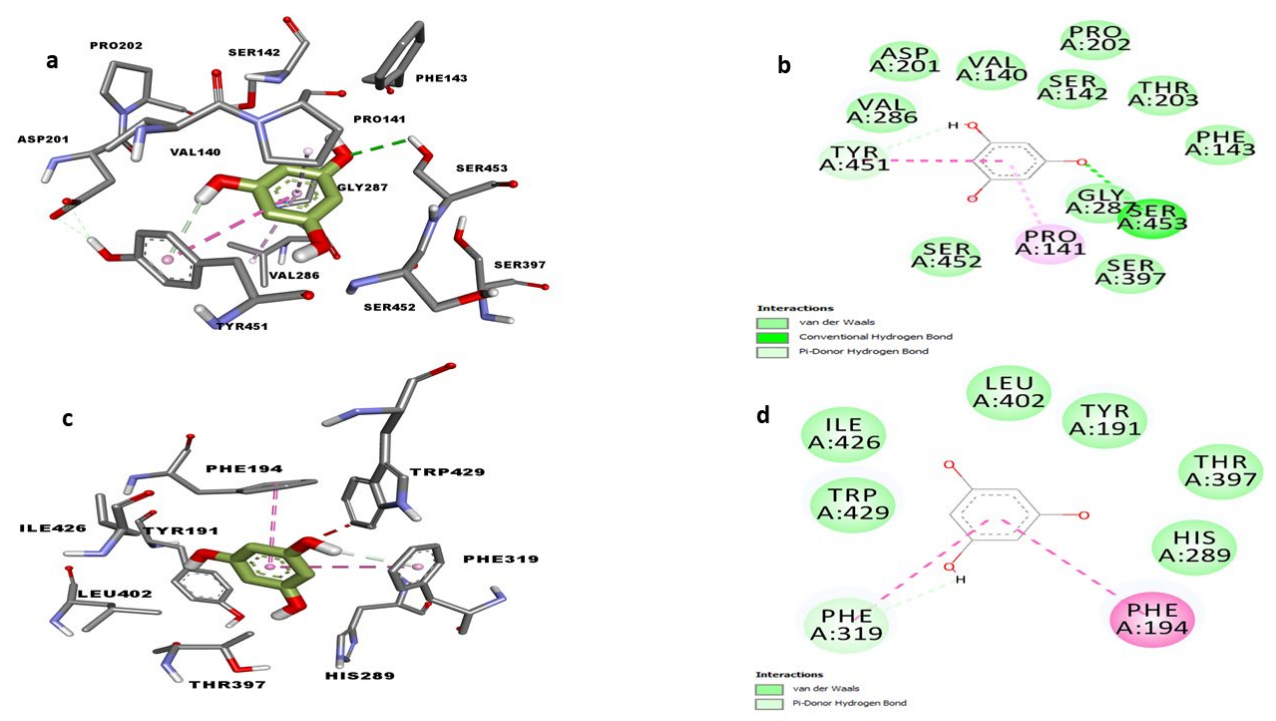
3D and 2D molecular interactions of phloroglucinol with trypanosomal sialidase (**a**) and (**b**) and PLA_2_ (**c**) and (**d**) after 50 ns molecular dynamics simulation studies.

**Figure 11:**
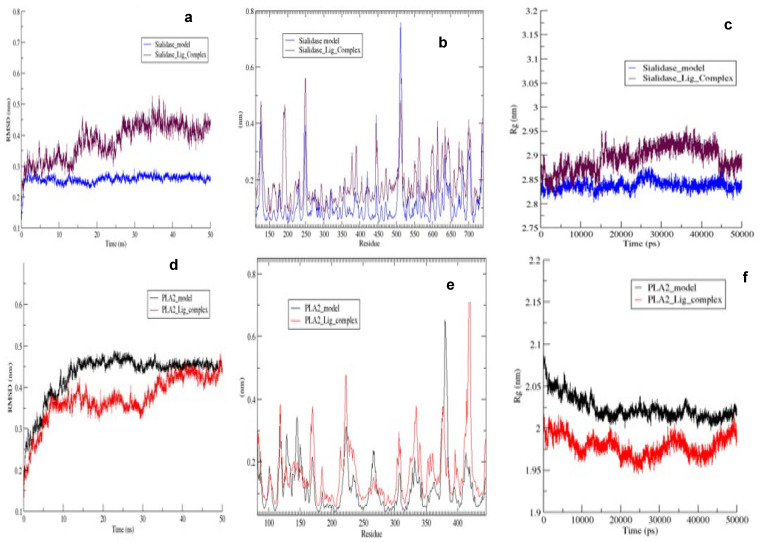
Molecular dynamics trajectory plots of unbound modelled trypanosomal sialidase and PLA_2_ in complex with phloroglucinol. RMSD (**a**) and (**d**), RMSF (**b**) and (**e**), ROG (**c**) and (**f**).

After establishing the in vitro effects of phloroglucinol on both *T. congolense* sialidase and PLA_2,_ molecular docking and molecular dynamics simulation studies were conducted. The optimized modelled structures of *T. brucei* sialidase and PLA_2_ had 97.9% and 97.2% of all their residues in the most favorable region respectively, whereas 0.6% and 1.6% were also in the additional allowed region but 1.5% and 1.3% of the residues were in the disallowed region (Figure 8A,B). Subsequently, phloroglucinol was docked against the simulated sialidase and PLA_2_, resulting to binding affinity values of −4.9 and −5.3 kcal/mol respectively. Visualization of the sialidase-phloroglucinol docked complex revealed two hydrogen bond interactions with Arg360 and Ser395 as well as, van der Waals and amide pi-stacked interaction with Gly396 and pi-anion with Glu337 (Figure 9b). Conversely, the compound interacted with PLA_2_ via a single hydrogen bond with Tyr191, van der Waals with Pro144 and pi-sigma with Val403 (Figure 9d). Further visualization of the interaction of phloroglucinol with modelled sialidase after the dynamics simulation showed the possibility of the compound to interact via van der Waal’s interactions, pi-alkyl, pi T-stacked and H-bond with Tyr451, Pro141, Tyr141 and Ser453 residues within the enzyme’s binding pocket (Figure 10a,c) while modelled PLA_2_ spanned only two pi-pi T-shaped interactions with Phe319 and Phe194 (Figure 10b,d). Thereafter, the stabilities of the enzymes were determined by 50 ns molecular dynamics simulation. The RMSD of the modelled trypanosomal sialidase in complex with phloroglucinol showed fluctuations from the beginning of the simulation with a steady rise up to 4.5 nm, with the unbound being more stable with lower RMSD values (Figure 11a). However, the unbound-PLA_2_ showed a rise to 0.3 nm from the beginning of the simulation till 10 ns from where it stabilized at an average of 3.6 nm until it reaches 30 ns before it fluctuated between 3.7–4.2 nm to the end of the simulation but the phloroglucinol-PLA_2_ complex attained more stability with lower RMSD values (Figure 11d). Similarly, low fluctuations were observed from the unbound trypanosomal sialidase residues (Figure 11b) while in the case of trypanosomal PLA_2,_ such fluctuations were mainly observed among the PLA_2_ residues involved in interaction with the phloroglucinol (Figure 11e). Moreover, radius of gyration (Rg) of the trypanosomal sialidase complex with phloroglucinol showed a higher value compared to the unbound model (Figure 11c) while lower Rg value was observed for PLA_2_-phloroglucinol complex in comparison to the modelled unbound PLA_2_ (Figure 11f).

Estimation of the free energies of binding using the molecular mechanics-Poisson-Boltzmann surface area (MM-PBSA) method revealed −67.84 ± 0.50 kJ/mol and −77.17 ± 0.52 kJ/mol for trypanosomal sialidase-phloroglucinol and PLA_2_-phloroglucinol complexes, respectively (Table 2). Other energies such as electrostatic energy, SASA energy, van der Waals energy, polar solvation energy are also shown in Table 2. Clustering analysis identified the most abundant structure accounting for 69% and 66% of the total conformations for sialidase and PLA_2_ complex with phloroglucinol.

The in silico ADMET prediction of phloroglucinol revealed the compound to be non-toxic with moderate half-life and clearance (Table S1). The compound was predicted to cross the blood brain barrier with optimal distribution. Moreover, phloroglucinol was predicted not to inhibit P-glycoprotein (Pgp) and the cytochrome enzymes, particularly CYP 450 1A2 and 3A4 (Table S1).

## 3. Discussion

In spite of the detrimental consequences of AAT and pathogenic effects of *T. congolense*, especially with respect to anemia development in animals, scientific attentions along these lines are limited. Herein, we demonstrated the chemotherapeutic potentials of phloroglucinol against *T. congolense* infection with emphasis on anemia amelioration using in vitro inhibition of sialidase and PLA_2_ as well as molecular docking and molecular dynamic simulations.

The treatment of *T. congolense* infected animals commenced after the appearance of *T. congolense* on day 4 pi but phloroglucinol was able to retard *T. congolense* proliferation mainly during the last 3 days of the treatment whilst complete elimination of the parasite from the bloodstream was not achieved. This is contrary to our previous observation where the phloroglucinol-rich fraction of *K. senegalensis* extract eliminated the parasites below detectable levels [10]. These two findings indicate that phloroglucinol might not be the only antitrypanosomal compound of the fraction. Meanwhile, the observed late trypanostatic effect of phloroglucinol might indicate the compound requires longer period of administration before it exerts the antitrypanosomal effects. An appealing observation was the positive effect of phloroglucinol in reducing animal mortality amidst infection. The zero mortality recorded in ITPGN30 group could indicate the possibility of the compound to increase animal’s endurance and responsiveness to the disease state [27]. Meanwhile, the parasitemia in the ITPGN30 group was increased but the level was far below the parasitemia profile of IC and ITPGN15 groups and hence, the lowered parasitemia in the ITPGN30 group could account for the increased survival of the animals in the group. This could be an important observation since death associated with Nagana is prominent in infected animal [12]. Moreover, the observed effect could be linked to immuno-modulatory activities of the phloroglucinol because previous studies have shown that the compounds can boost immunity and induce resistance of shrimps against aquatic pathogen [28]. On the other hand, the basis for the observed higher mortality in the ITPGN15 group compared to the IC group is not clear at the moment. However, it may be possible to link the observation to the inability of the dosage to stimulate the immunity of the animals.

Most often, mortality associated with the AAT are associated with anemia development and organs destruction which are considered as major pathological features associated with the *T. congolense* infection [6,24] and they were prominent in our study. With respect to organ damage, only splenomegaly and renal damage induced by *T. congolense* were reversed by the phloroglucinol (30 mg/kg BW) and this could be an important finding because splenomegaly and kidney enlargement have been reported as additional factors contributing to anemia development during the infection [29]. Indeed, this might be linked to the observed amelioration of anemia to almost baseline value by the 30 mg/kg BW treatment with phloroglucinol.

The involvement of sialidase as a key player in anemia development [11,12] in *T. congolense* was investigated by monitoring the levels of cleaved sialic acid from the surface of host erythrocyte membrane in addition to in vitro inhibition of the enzyme. It was interesting to observe that phloroglucinol inhibited the bloodstream *T. congolense* sialidase activity of the enzyme via mixed inhibition pattern suggesting interaction with the pure enzyme as well as the sialidase-fetuin complex. Further, the observed Ki of 0.181 µM was appreciably lower than the Ki values of ellagic acid, stigmasterol, geranylacetone and phytol against the same enzyme [6,19,20,21] suggesting that the compound might be a highly promising candidate as *T. congolense* sialidase inhibitor. In addition to inhibition of sialidase, the compound also inhibited PLA_2_ which has also been implicated in anemia development during the infection [9] but, no pronounced effect was observed since the Ki value was >9000 µM suggesting the compound is more active against *T. congolense* sialidase than PLA_2_.

Molecular docking and molecular simulation dynamics were conducted to determine the interactions and stability of the compound with trypanosomal sialidase and PLA_2_. Interestingly, the compound interacted with the sialidase and PLA_2_ with fairly good binding energy but higher than geranylacetone and phytol which apparently did not agree with the in vitro observations. It is plausible that the relatively higher hydrophilicity of the phloroglucinol (compared to phytol and geranylacetone) makes it easier for the compound to interact with the enzyme in our in vitro reaction milieu. Additionally, the Arg360, Ser395, Gly396, Tyr451, Ser452, and Pro141 residues involved in the interactions were critical active site residues (Figure S1) as depicted by FTsite server. In the case of PLA_2_, the Ala185, Tyr194 and Val403 that participated in the interaction were also critical active site residues (Figure S2). Molecular dynamics simulation conducted at 50 ns showed sialidase-phloroglucinol complex with low RMSD to be more stable compared to PLA_2_-phloroglucinol complex although the latter was more compacted, exhibiting higher fluctuations. Meanwhile, it is known that stability and compactness of ligands within the enzyme is a measure of excellent structural complementarity [30]. Furthermore, a consistent SASA value was observed with the complexes and hydrogen bond interaction with Ser453 residue of sialidase in addition to higher binding energy compared with PLA_2_. However, unfavorable contribution from solvation energy was higher in the sialidase-phloroglucinol complex than the PLA_2_-phloroglucinol complex. Overall, it seems that the molecular dynamic trajectories somehow favor PLA_2_ but our in vitro inhibition kinetics have proved otherwise. It is possible that the presence of hydrogen bond interaction with high stability of phloroglucinol on sialidase might have been translated to the observed lower Ki value and the possible anti-sialidase activity of the compound observed in our in vitro kinetic studies. Additionally, conducting the molecular dynamic simulation for a longer period such as 100 ns with a positive control could further clarify the significance of these results. Meanwhile, in silico ADMET calculations predicted phloroglucinol to be non-toxic. Most often, the in silico ADMET predict the success of lead compounds and interestingly, the compound appeared not to be hepatotoxic and predicted not to inhibit the function of important cytochrome enzymes needed for the detoxification of foreign substances.

## 4. Materials and Methods

### 4.1. Chemicals and Reagents

Phloroglucinol, fetuin and *N*-acetylneuraminic acid were obtained from Sigma Chemical Company (Saint Louis, MO, USA) while sodium periodate and thiobarbituric acid (TBA) were obtained from KEM light laboratories PVT Ltd., India. Diethylaminoethyl (DEAE) cellulose and sodium arsenate were obtained from SISCO research laboratories Ltd., India and Lab Tech Chemicals, respectively. Assay kits for the evaluation of serum aspartate and alanine aminotransferases (AST and ALT), urea and creatinine were procured from LABKIT (Chemelex, S.A., Barcelona, Spain) and diminazine aceturate (DA) was purchased from a local veterinary clinic in Zaria but was produced by Eagle Chemical Company Ltd., Ikeja, Nigeria.

### 4.2. Experimental Animals and Source of the Trypanosomes

Apparently healthy Wistar rats of both sexes weighing between 150–200 g were purchased from Department of Pharmacology and Therapeutics, Ahmadu Bello University, Zaria- Nigeria. The animals were handled according to the guidelines of Good Laboratory Practice regulations of the World Health Organization (WHO) with the ethical approval from Ahmadu Bello University Committee on Animal Use and Care (ABUCAUC); approval number ABUCAUC/2018/006. Additionally, the study was conducted and reported according to the ARRIVE guidelines (https://arriveguidelines.org/) (accessed on 30 September 2021). The animals were fed with commercial rat chow (ECWA Feeds, Jos, Nigeria) and water was provided ad libitum. The *T. congolense* (Savannah strain) used in the study was obtained from the stabilates at the National Trypanosomiasis and Onchocerciasis Research (NITOR), Kaduna-Nigeria.

### 4.3. Animal Grouping and Evaluation of the In Vivo Anti-T. congolense Effect of Phloroglucinol

For the in vivo study, a total of thirty five (35) rats were randomly divided into five (5) groups of seven (7) rats each according to the following design: Normal control (NC) group where the animals were neither infected nor treated and Infected control (IC) group where the animals were infected but not treated. Two other groups were infected and treated with 15 (ITPGN15) and 30 mg/kg BW (ITPGN30) of phloroglucinol while the remaining group of animals was treated with 3.5 mg/kg BW diminazene aceturate (ITDA). After grouping, the rats in the infected groups were intraperitoneally injected with 0.4 mL/100 g of 10^4^ parasites/mL of the infected blood, diluted in cold phosphate buffer saline. On the fourth day after infection (4 pi) when parasites were detected in the blood of the infected animals, daily treatment was initiated with phloroglucinol and diminazene aceturate through oral and intra-peritoneal routes, respectively. The daily treatment was sustained for 14 days while animals in NC and IC received the vehicle only. In the infected groups, daily parasitemia was also monitored using the rapid matching counting method and percentage animal survival was determined by counting the mortality in each group every day. Additionally, post and pre-infection packed cell volume (PCV) of all the experimental animals were determined at day 0 and day 14 (termination day of the study) using the micro-hematocrit method and the % change in PCV was computed.

### 4.4. Collection of Blood and Organ Samples

On the final day of the experiment, the experimental animals were euthanized using chloroform anesthesia and blood samples were collected in plain containers via cardiac puncture. The collected blood samples were allowed to settle and finally centrifuged at 3000× *g* for 15 min. The serum was collected in fresh containers and stored at −30 °C for biochemical analyses as well as estimation of free serum sialic acid levels. The liver, kidney, spleen and brain of the experimental animals were immediately removed after sacrifice, washed with normal saline, wiped with filter paper and weighted to ascertain the relative organs weights of the animals. For histopathology studies, the organs were immediately placed in 10% formalin in clean containers and stored until needed for analysis.

### 4.5. Assessment of Hepatic and Renal Function and Free Serum Sialic Acid Level

Serum activities of alanine aminotransferase (ALT) and aspartate aminotransferase (AST) were used as indices of hepatic function while serum levels of urea and creatinine were considered as markers of renal function which were evaluated using commercially available reagent (LABKIT) following the manufacturer’s protocols. On the other hand, free serum sialic acid levels of the experimental animals were determined using the TBA assay. Briefly, 200 µL of serum was mixed with 100 µL of 25 mM periodate solution and allowed to stand at 37 °C for 30 min before the addition of 2% sodium arsenate (200 µL). The mixture was capped and then mixed thoroughly before the addition of 2 mL of 0.1 M TBA after which the tubes were heated at 80 °C for 8 min. Thereafter, the samples were cooled for 5 min and 2.5 mL of acid-butanol reagent was added and centrifuged at 3000× *g* rpm for 5 min. The absorbance of the butanol layer was measured at 549 nm and the sialic acid concentration of the samples was determined from a sialic acid standard curve.

### 4.6. Inhibitory Effects of Phloroglucinol against Trypanosoma congolense Sialidase and PLA_2_

Bloodstream *T. congolense* sialidase and PLA_2_ that were isolated and partially purified on a DEAE cellulose column in our laboratory were used in this study. The specific activity of the *T. congolense* sialidase was 2.27 µmol/min/mg while the PLA_2_ had a specific activity of 39.26 µmol/min/mg. The sialidase activity was routinely determined using previously described methods [31] where equal volume (500 µL) of 100 mg/mL of fetuin (dissolved in 50 mL of 0.2 M sodium citrate phosphate buffer pH 6.7) and enzyme (partially purified sialidase) were mixed and incubated at 37 °C for 30 min. The reaction was stopped by the addition of 250 µL of periodate solution and the released sialic acid was quantified using TBA method as earlier described. The activity of sialidase was defined as the amount of enzyme which catalyzed the hydrolysis of 1 µmol sialic acid per minute from fetuin. In the case of PLA_2,_ the egg yolk coagulation method of Gomes and Hannahpep [32] was used for the routine assay where 500 µL of lyophilized egg yolk (1 mg/mL) was mixed with 50 µL of calcium chloride solution, 100 µL of enzyme solution (in phosphate buffer pH 7.2) and the mixture was incubated at 37 °C for 10 min. The reaction was terminated by heating the reaction mixture at 100 °C for 1 min and the amount of the released free fatty acid was determined titrimetrically at pH 8.0 using 20 mM NaOH. The PLA_2_ activity was defined as the amount of enzyme that catalyzed the released of 1 µmol of free fatty acid per minute. Total protein was also determined using the Lowry’s method with bovine serum albumin (BSA) as the standard.

The inhibition study was conducted by assaying the activities of sialidase and PLA_2_ using variable concentrations of phloroglucinol. Data on the initial rates of the sialidase and PLA_2_ activities were presented graphically using double reciprocal plot and Michaelis Menten’s constant (K_M_), maximum velocity (Vmax) and inhibition binding constant (K_i_) were determined.

### 4.7. Sequences Retrieval and Homology Modelling of T. congolense Sialidase and PLA_2_

The putative sequences of sialidase (TcIL3000.A.H_000336800) and PLA_2_ (TcIL3000_0_00740) retrieved from TriTrypDB (https://tritrypdb.org/) (accessed 16 March 2021) were shown to have a high similarity index with the *T. brucei* homologues and hence, the amino sequences of the *T. brucei* sialidase (Q57TY5) and PLA_2_ (AEX60759.1) were retrieved from UniProt Knowledgebase database (http://www.uniprot.org/) (accessed 19 March 2021) and NCBI (https://www.ncbi.nlm.nih.gov/) (accessed 19 March 2021) respectively. Subsequently, the active residues at the binding sites of the proteins were determined by FTSite binding site prediction server (https://ftsite.bu.edu) (accessed 21 April 2021). Interestingly, these residues were further confirmed to be conserved in both *T. brucei* and *T. congolense* respectively during multiple sequence alignment in ClustalW server (https://www.ebi.ac.uk/Tools/services/rest/clustalo) (accessed 25 April 2021) (Figures S1 and S2). After these confirmations, the structures of *T. brucei* sialidase and PLA_2_ were modelled using SWISS-MODEL server based on the crystal structures of *T. rangeli* sialidase in complex with 2,3-difluorosialic acid (PDB ID; 2A75) and Not1 C-terminal domain in complex with Not4 (PDB ID; 5AJD) respectively. The SWISS-MODEL server was used for the modeling because it generated better output compared to Modeller after series of model production. Overall, the template structures were selected from protein BLAST against protein data bank (PDB) based on high sequence identity, sequence length, high resolution, domain coverage and E-value (Tables S2 and S3). Thereafter, the predicted structures were subjected to molecular dynamics simulation for further structural optimization and determination of their conformation in real biological system. The molecular dynamics simulation was performed in three stages with GROMACS [33] and OPLS-AA force field used for the optimization of the protein structure in a box of water, while the topology files for the modeled proteins were prepared by the ‘pdb2 gmx’ script. The first stage of the molecular dynamics simulation was to eliminate major atomic clashes in the proteins where minimization stage was achieved by employing the steepest descent minimization algorithm for both protein models. For the PLA_2_ protein model, steepest descents converged to Fmax < 1000 in 1278 steps with the potential energy of the system estimated to be −1.3787904 × 10^6^ and the maximum force being 9.2160840 × 10^2^ on atom 2981 while for the sialidase model, steepest descents converged to Fmax < 1000 in 1960 steps with the potential energy of the system estimated to be −2.8664222 × 10^6^ and the maximum force being 9.6007135 × 10^2^ on atom 697. After minimizing the systems, the system was equilibrated (conducted in two phases). The first phase of equilibration was conducted under an NVT ensemble, using a leap-frog integrator at a time step of 2 fs and 50,000 steps for a total of 100 ps, the system reached equilibrium as the temperature reached a plateau at 300 K. In the second phase of the equilibration, the system was equilibrated and conducted under an NPT ensemble using the Parrinello-Rahman barostat for pressure coupling. The leap frog integrator was also used for the pressure equilibration at a time step of 2 fs and 50,000 steps for a total of 100 ps. Lastly, the production stage was performed without position restraints for a total of 50 ns with a time step of 2 fs at 310 K in NPT ensemble. The resulting trajectory was visualized and analyzed with VMD and GROMACS. The simulated proteins were extracted in a pdb format after clustering, using the gmx cluster module in the GROMACS (2018.3) software with RMSD cut-off of 0.2 nm (backbone) and were further evaluated to determine their stereochemical quality (bonds angles, phi (Φ) and psi (ψ) torsion angles, non-bonded atom-atom distances and dihedral angles) using the Ramachandran plot of the PROCHECK program in Structure Analysis and Verification Server (SAVES) v5.0 meta server (UCLA MBI) (https://saves.mbi.ucla.edu/) (accessed 14 May 2021).

### 4.8. Molecular Docking of Phloroglucinol against Modelled Structures

The molecular docking of the phloroglucinol in sdf format from pubchem database (https://pubchem.ncbi.nlm.nih.gov/) (accessed 16 March 2021) against the modelled sialidase and PLA_2_ was performed using Autodock Vina [34]. Initially, both enzymes and ligand were minimized using MMFF94 force field [35] and were subsequently converted to pdbqt files before the docking grids were finally defined by selecting the specific area around the catalytic residues for *T. brucei* sialidase (X: 9.2231; Y: −10.301; Z: 19.3286) as well as *T. brucei* PLA_2_ (X: −10.503; Y: −2.946; Z: −5.598). At the end of the docking studies, binding energy (kcal/mol) of the enzymes in complex with phloroglucinol were computed.

### 4.9. Molecular Dynamics (MD) Simulations and Energy Calculation

To elucidate the conformational stability, dynamics, structural stability, folding properties and compactness of sialidase-phloroglucinol and PLA_2_-phloroglucinol complexes, MD simulations were performed for a period of 50 ns using GROMACS as previously described [36]. The protein-ligand complexes were also minimized and equilibrated in two stages following the pattern used for the protein models. The gromos54a7 force field was used to generate the protein topology and the PRODRG server was used to generate the topology and parameters for the ligand. The sialidase-phloroglucinol complex system was minimized by steepest descents which converged to Fmax < 1000 in 619 steps with the potential energy of the system estimated to be −1.8670971 × 10^6^ and the maximum force of 9.0477020 × 10^2^ on atom 3898. Likewise, the PLA_2__phloroglucinol complex system was minimized by steepest descents which converged to Fmax < 1000 in 288 steps with the potential energy of the system estimated to be −6.7606069 × 10^5^ and the maximum force was 9.1669098 × 10^2^ on atom 2829. The two protein ligand complexes were equilibrated in two phases. The first phase was conducted under an NVT ensemble, using a leap-frog integrator at a time step of 2 fs and 50,000 steps for a total of 100 ps while the system reached equilibrium as the temperature climaxed at 300 K. In the second phase of the equilibration, the system was equilibrated and conducted under an NPT ensemble using the Berendsen barostat for pressure coupling. The leap frog integrator was also used for the pressure equilibration at a time step of 2 fs and 50,000 steps for a total of 100 ps. Additionally, geometric clustering was performed from the 50 ns simulation trajectories of the protein-ligand complexes using the gmx cluster module in the GROMACS (2018.3) software where RMSD cut off of 0.2 nm (backbone) was used to select representative structures for each cluster. The binding free energies of the modelled enzymes in complex with phloroglucinol were calculated using the Mechanic/Poisson-Boltzmann Surface Area (MM-PBSA) equation [37] as shown below;

Δ*G binding* = Δ*G complex* − (Δ*G protein* + Δ*G ligand*) (1)

where, Δ*G binding* signifies the total energy of binding of the protein-ligand complex while Δ*G protein* and Δ*G ligand* represent the energies of the free receptor and unbound ligand respectively.

### 4.10. In Silico ADMET Prediction of Phloroglucinol

In order to gain insights into the pharmacokinetics and pharmacodynamics parameters of phloroglucinol, ADMETLab (http://admet.scbdd.com/) (accessed on 8 November 2021) was used to determine some properties of the compound. The smiley of phloroglucinol retrieved from PubChem database was uploaded in the ADMETLab. Thereafter, the compound was subjected to a systematic ADMET evaluation where, log P and log S as an index of physicochemical properties in addition, HIA, F30 availability, Pgp-inhibitor, plasma protein binding, blood brain barrier, CYP450 3A4, half-life, clearance, human hepatotoxicity parameters were predicted.

## 5. Conclusions

In conclusion, our findings have demonstrated that phloroglucinol could suppress the proliferation of bloodstream form of *T. congolense* but complete elimination of the parasites was not achieved. However, the compound is active at ameliorating the *T. congolense* induced anemia which could be mediated mainly through inhibition of bloodstream *T. congolense* sialidase and not PLA_2_.

## Figures and Tables

**Table 1:** Effects of oral treatment with phloroglucinol on biochemical parameters and relative organs weight of *T. congolense*-infected rats.

Biochemical Parameters/ Relative Organs Weight	Normal Control	Infected Control	ITDA	ITPGN15	ITPGN30
Alanine aminotransferase (U/L)	1.94 ± 1.34 ^a^	2.72 ± 0.67 ^a^	7.58 ± 4.12 ^b^	31.5 ± 2.33 ^c^	12.25 ± 0.82 ^b^
Aspartate aminotransferase (U/L)	1.74 ± 0.83 ^a^	2.04 ± 1.24 ^a^	2.91 ± 0.58 ^a^	4.08 ± 0.01 ^b^	1.51 ± 0.88 ^a^
Urea (mg/dL)	36.61 ± 1.74 ^a^	43.44 ± 1.34 ^b^	50.00 ± 3.92 ^c^	54.62 ± 3.92 ^c^	41.42 ± 1.83 ^b^
Creatinine (mg/dL)	0.99 ± 0.07 ^a^	2.02 ± 0.47 ^b^	2.44 ± 0.87 ^b^	20.70 ± 0.08 ^c^	3.98 ± 1.57 ^b^
Relative liver weight (%)	2.70 ± 1.08 ^a^	4.77 ± 1.58 ^a^	2.71 ± 0.27 ^a^	3.75 ± 1.65 ^a^	2.76 ± 0.44 ^a^
Relative kidney weight (%)	0.80 ± 0.37 ^a^	2.67 ± 1.27 ^b^	0.71 ± 0.21 ^a^	1.70 ± 0.22 ^b^	0.47 ± 0.15 ^a^
Relative spleen weight (%)	0.48 ± 0.11 ^a^	2.95 ± 1.69 ^c^	0.70 ± 0.35 ^a^	1.12 ± 0.04 ^b^	0.82 ± 0.15 ^a^
Relative brain weight (%)	0.85 ± 0.34 ^a^	2.15 ± 1.03 ^a^	0.89 ± 0.19 ^a^	0.98 ± 0.16 ^a^	0.71 ± 0.15 ^a^
Free serum sialic acid (mg/mL)	2.83 ± 0.61 ^c^	2.98 ± 0.02 ^c^	0.84 ± 0.04 ^a^	1.14 ± 0.01 ^b^	3.52 ± 0.20 ^c^

All data are presented as mean ± standard deviation of seven rats. Data was analyzed using ONE-WAY ANOVA followed by (Tukey’s multiple range post-hoc test). Values with different subscripts (a, b or c) between groups are considered statistically significant at *p* < 0.05. ITPGN15 = Infected + 15 mg/kg BW PGN, ITPGN30 = Infected + 30 mg/kg BW PGN, ITDA = Infected + 3.5 mg/kg BW DA.

**Table 2:** MM-PBSA calculations of binding energy for phloroglucinol docked to sialidase and PLA_2_.

Docking Complexes	Binding Free Energy (kJ/mol)	SASA (kJ/mol)	Polar Solvation (kJ/mol)	Electrostatic (kJ/mol)	Van der Waal (kJ/mol)
Sialidase-phloroglucinol	−67.84 ± 0.50	−9.132 ± 0.027	36.363 ± 0.304	−1.209 ± 0.050	−93.881 ± 0.456
PLA_2_-phloroglucinol	−77.17 ± 0.52	−9.233 ± 0.030	24.304 ± 0.248	−0.543 ± 0.044	−91.670 ± 0.504

## Data Availability

Not applicable.

## Supplementary Materials

The following are available online. Figure S1: Alignment of *T. congolense* sialidase with *T. brucei* sialidase. Figure S2: Alignment of *T. congolense* PLA_2_ with *T. brucei* PLA_2_. Table S1: Drug likeness and ADMET prediction of phloroglucinol. Table S2: BLAST and alignment details of target templates for the selection of 3D structure for modeling of sialidase. Table S3: BLAST and alignment details of target templates for the selection of 3D structure for modeling of phospholipase A_2_.

## Sample Availability

Samples of the compounds are not available from the authors.
