# Supplementary material for: Phloroglucinol as a Potential Candidate against Trypanosoma congolense Infection: Insights from In Vivo, In Vitro, Molecular Docking and Molecular Dynamic Simulation Analyses
Source: Molecules. 2022 Jan 12;27(2):469. doi: 10.3390/molecules27020469 (PMC8781988; doi:10.3390/molecules27020469)
Supplement: Supplementary file 1 [file molecules-27-00469-s001.zip › molecules-1476885-supplementary.pdf]

SUPPLEMENTARY MATERIALS

Phloroglucinol as a Potential Candidate against Trypanosoma Congolense Infection: Insights from In Vivo, In Vitro, Molecular Docking and Molecular Dynamic Simulation Analyses

Nasirudeen Idowu Abdulrashid [1], Suleiman Aminu [1], Rahma Muhammad Adamu [2], Nasir Tajuddeen [3], Murtala Bindawa Isah [4], Isa Danladi Jatau [5], Abubakar Babando Aliyu [3], Mthokozisi Blessing Cedric Simelane [6], Elewechi Onyike [1] and Mohammed Auwal Ibrahim [1,*]

1 Department of Biochemistry, Ahmadu Bello University, Zaria 810241, Nigeria; abdulrashidmuhammadnasir@gmail.com (N.I.A.); suleimanaminu@abu.edu.ng (S.A.); elewechionyike@gmail.com (E.O.)
2 Department of Biotechnology, School of Engineering and Technology, Sharda University, Greater Noida 201310, India; turanjoji84@gmail.com
3 Department of Chemistry, Ahmadu Bello University, Zaria 810241, Nigeria; ntajuddeen@yahoo.com (N.T.); aliyubabando@gmail.com (A.B.A.)
4 Department of Biochemistry, Umaru Musa Yar'adua University, Katsina 820241, Nigeria; isah.murtala@umyu.edu.ng
5 Department of Veterinary Parasitology and Entomology, Ahmadu Bello University, Zaria 810241, Nigeria; mail4idjatau@gmail.com
6 Department of Biochemistry, University of Johannesburg, Johannesburg 2001, South Africa; msimelane@uj.ac.za
* Correspondence: mauwalibrahim@gmail.com or maibrahim@abu.edu.ng; Tel.: +234-7031104932

Figure S1: Alignment of *T. congolense* sialidase with *T. brucei* sialidase

```
congolense    ------------MMGYSKSVRQTLICLLLVAIDTYHCTTAYGSEIKGEEEENRSLFLPG          47
brucei          MERLQVRMGTLSLTSPFSMLAYPFLPCTVVILSVLSFCCGIVSS--KIYEKTTREVFLEG        58
                          :* .  ** :::    .*    .*  *  *: .*.:** *

congolense GLWYKKDEWKDGNWLQSKEWKAGYAWWPWRSWCSNKTIGETVKELCRKEWDSQRAKGYTL  107
brucei      GRWVRKSEWEKGSWKTSPEWNAGYEWWA---WCMDSVAKEAKGEVCRKEWLSQRKKGYTL   115
             * * :*.**:.*.*  * **:*** **    ** :..  *:  *:***** *** *****

Congolense   KPRESVLFRESSGTKRMRRVHSFRIPSMVEANGVLIGIADARYLSSADFTFIDTVAKYSA        167
brucei      VPRTKVPFREKNGTQWMRNVHSFRVPSFVEVSGVLVGIADVRYISSADFTFTETVAKYSA        175
              ** .* ***..**: **.*****:**:**..***:****.**:******* .:*******

congolense DGGETWKTEVIIENARVDSFHSRVVDPTVAVKNNSIYVLVGRYNTSKAYWTWQHYGNDWD      227
brucei      DGGDTWKTKVIIENSRVNTNFSRVVDPTVAVKGNNIFVLVGRYNTSSKYWTWQHYGEDWD      235
             ***:****:*****:**::.***********.*.*:*********.********:***

congolense ILMYKGTVTKTSEDGKPAANIEWTSVLNLKSLLETGLYVGGHEATQFLGGVGNTVVTPDG       287
brucei      ILMYKGTVIKEEKDGNVTASITFEAPQNLKFLLATVPSPGGHPPSQFLGGVGNAAVTPDG 295
             ******** *  ..**: .:*.* : :  *** ** *    ***  .********..*****

congolense TIVFPIQVKNSWNHVAAMIMYSSDDGATWHLGGGATPVGTTEASAIWWDGKLVLNCRT--       345
brucei      AIVFSVQVKNTWNHVVGKLLYSTDDGKTWHFGAGETPVGSTESSVVWWKDRLLVNARTAE      355
              :*** .****:****.. .:**:*** ***:*.* ****:**:*.:**..:*:.*.**

Congolense   DLGYRKVFETTDLGTTWKESLGALSRVIGNSPDRKQPGSSGSAITLEVEGVQVMLITQPK       405
brucei      YVGYRRVFETSDLGNTLKESISTLSRVIGNSPLRNQPGSSGSAISITVEGMDVMLISQPK        415
               :***:****:***.* ***..:********* *.*********:: ***::****:***

congolense NTKTRYSRDRLQLWLSDGSRVWLVGQISRGNDNSPYSSLLYTSDDKLYCLYEQNIEEVYS       465
brucei      NEKGSFSRDHLQLWLTDGTRVFLVGQISQGDDNSPYSSLLYTSDGKLYCLYEQKIEEVFT475
              * *  :***:*****:**:**:******:*:************* ********:****::

congolense IYLVHLVDELEKIKATVRLWKEQDALLSGNCSATAEDGSDCNGVPTAGLVGLLSGPAQGN       525
brucei      IYLARLVDEMKMIKRVVLLWKAQDALLVGDCLSSVGGTRPCKGIPVGDLAGLLTGPAVGH      535
             ***.:****:: ** .* *** ***** *:* ::.    *:*:*...*.***:*** *:

congolense AWPDAYNCVNASLVNVTSDADGLQLGGLNRGRVSWPVRAQGQDQRYYFANVRFTLVATVQ      585
brucei      VWPDVYKCVNASVSGAVDNKRGVVLGGTSGSSVVWPVSEQGQDQRYYFANTHFTIVATVQ      595
              .***.*:*****: ....:  *: *** . . * ***  ***********.:**:*****

congolense LMKAPNSNVAVLGFGNSKGENLT---LWVASTTWTLTYGGERKKVVAPSLSSDASVQFAL       642
brucei      FGAVPQRDTPLIGFVNGEKNANKLFILSIENTRWTLMYGEKCSEGSPVPSNLEETHQIAL  655
                 :  .*: :. ::** *.: :  .    * : .* *** ** : .:      . : : *:**

congolense  ILNGGSVSVYADGVH-VPQLDKRVAAKNKLLNIDHFFAESNY---MGDTNNIFTKNMLLY 698
brucei      ALQDGLVVAYVDGKLAVAAINVSESDRVDLLNIRHFFVGTPASSDLSDHTSITVHSVLLY      715
                        *:.* * .*.**   *  ::   : : .**** ***. :     :.* ..* .:.:***

congolense NRKLSESELKLLSLNREAIRAADGLNYLKEQQGGSESEIKSTSD-------SNVS----D 747
brucei      NRRLSEGELQLVFTNREVIRAANPTTPLPVSSRVAAGAGRQSHDGVFFTFGDHVSSVRSR 775
             **:***.**:*:  ***.****:  . *  ..  : .  .:: *       .:**

congolense PADNETSEKMFLQV-------AL-ILLLVIGQD--------        772
brucei      YSDGGILEYVYLLSVISLCALTLFILTLVFQRRRDVVPTNI     816
```

"Amino acid residues: Arg360, Ser395, Gly396, Tyr451, Ser452, Ser453 and Pro141 involved in the interaction of the Sialidase docked to phloroglucinol before and after the 50 ns MD simulation

were found conserved in both parasites. These residues were also found to be critical as active residues in the binding site of the protein determined from the FTSite server”.

Figure S2: Alignment of *T. congolense* PLA_2 with *T. brucei* PLA_2

```
congolense MPFWTQKDVARLVRWSTESFFLGHSLPLRGRLTSLHFAPVSVALVSSFVMYLNGMSSMWS      60
brucei      MVTWALKYFVRVVRWSTEAFLIWPTRPLFDYATSLHCVPISGTFITSVLLCFYGFPLLWS 60
             *  *: * ..*:******:*::  : ** .  **** .*:* ::::*.:: : *:  :**

congolense SLAVCIGVLVSGGMFIVFPLPVLRPIGGRYCVSIAHMDGRASQQLPPVAVFYPTNMSPDI  120
brucei      TAMVAVGFIISGVLFLVSPLPLLKPIGGRYSVGLVHMNGCRSQSIPPVAVFYPTNMVPEK  120
          :   *.:*.::** :*:* ***:*:******.*.:.**:*  **.:*********** *:

congolense KGISYVPFDDSRFIRGVAAYAKLPYFFLKDFMLLRVGATMNAVQADILS-NGRMPPIVVF  179
brucei      KGLPYVPFGDDRFLRGVAAYANVPFFFIRDFSFVRISASRNAVPAALLNQYERVPPIVVF  180
             **: ****.*.**:*******::*:**::** ::*:.*: *** * :*.   *:******

congolense SHGLAGYHRLYSCFAMDLAARGAVVVSVGHCDNSASFMRDSTGEESAVYLKDYGWQVPKR      239
brucei      SHGLAGYHLFYSCFALDLAARGAIVICLGHCDNSASFMRDSSGKESEVPLKDYGWEVPAR      240
             ******** :*****:*******.*:.:*************:*:** * ******:** *

congolense EMQISKRVKEVRRVLERLGETDFWTDLGCSASDAENFLSRRPQVHLSGHSFGGATALAVA      299
brucei      EAQVAQRVSEVRGTLQRLTEKDFWTTLGYSNSDIDKFLSKPLQVHLAGHSFGGATVLAAA      300
             * *:::**.*** .*:** *.**** ** * ** ::***:  ****:********.**.*

congolense LEESQNATKVVNVRSVFVYDPWMLPIQGEHFFKPLSDGSKAYCIPTVTIHSDDWVNDSES359
brucei      LEENQNPVKGVSVKSVYTFDPWMVPIQNEHFCNPLSDGRKSYTVPTVTVHSDDWVKDSES      360
             ***.** .* *.*:**:.:****:***.*** :***** *:* :****:******:****

congolense  WSFFQKVKSVVLAQPSYASLGSDEREAKFRTLITRNTSHISMVDIAVLSPVIHGKVNSVV419
brucei      WEFFKRMKALVLEQSAYASLNEVEKQALFGIVVTKNTNHLSLVDVSVLSPVMHGNIWATV      420
              *.**:::*::** * :****.. *::* *  ::*:**.*:*:**::*****:**:: :.*

congolense SPRVQIMEWCNALLQFARANTVVSDLS    446
brucei      SPRVQIMEWCNALLRFAKQNTEVCSTC    447
             **************:**: ** *.. .
```

“Amino acid residues; Ala185, Tyr191, Tyr194 and Val403 involved in the interaction of the target proteinPLA_2 and phloroglucinol before and after the 50 ns MD simulation were found conserved in both the parasites. These residues were also found to be critical as active residues in the binding site of the protein determined from the FTSite server”.

Table S1. Drug likeness and ADMET prediction of phloroglucinol

**Phloroglucinol**	**Properties**
Molecular formula	C6H6O3
IUPAC name	**1,3,5-Benzenetriol**
Molecular weight	126.031693 Da
Octanol-water partition coefficient (logP)	0.313
Topological polar surface area (TPSA) Å^2	60.690
Hydrogen bond donors	3
Hydrogen bond acceptors	3
Number of rotatable bonds	0
Lipinski's rule	Accepted
Pfizer rule	Accepted
Pains	0 alert
Caco-2 Permeability	-4.791 (excellent)
Gastrointestinal absorption (GIA)	Excellent
Blood brain barrier (BBB)	Permeate (excellent)
P-glycoprotein (P-gp) substrate	Excellent
P-gp Inhibitor	Excellent
CYP1A2 inhibitor	Yes
CYP2C19 inhibitor	No
CYP2C9 inhibitor	No
CYP2D6 inhibitor	No
CYP3A4 inhibitor	Yes
Clearance (CL)	14.917 (Excellent)
hERG Blockers	None
AMES Toxicity	None
Rat Oral Acute Toxicity	None

Carcinogenicity	None

Table S2. BLAST and alignment details of target templates for the selection of 3D structure for modeling of sialidase

Template ID	Sequence identity	Resolution	Domain coverage	Sequence length	E-value
1MS5	43.06%	2 Å	75%	648	3e-153
1MR5	43.06%	2.25 Å	75%	648	4e-153
1WCS	41.60%	2.8 Å	75%	641	9e-153
3PJQ	42.90%	2.1 Å	75%	648	2e-152
1S0I	42.90%	1.6 Å	75%	648	5e-152
1N1S	41.03%	1.64 Å	75%	641	1e-148
**2A75**	**41.03%**	**1.95 Å**	**75%**	**652**	**3e-148**

Table S3. BLAST and alignment details of target templates for the selection of 3D structure for modeling of phospholipase A_2

Template ID	Sequence identity	Resolution	Domain coverage	Sequence length	E-value
6M07	31.21%	2.64 Å	34%	369	3e-13
6M06	31.21%	2.1 Å	34%	371	3e-13
**5AJD**	**31.21%**	**2.05 Å**	**34%**	**388**	**3e-13**
3D59	31.21%	1.5 Å	34%	383	3e-13
5I8P	31.21%	2.37 Å	34%	388	3e-13
3F96	31.21%	2.1 Å	34%	383	1e-12
